# The effect of two educational technology tools on student engagement in Chinese EFL courses

**DOI:** 10.1186/s41239-021-00263-0

**Published:** 2021-05-28

**Authors:** Yilian Teng, Xia Wang

**Affiliations:** School of Foreign Studies, Beijing Information Science & Technology University (BISTU), 12 Xiaoying East Road, Haidian District, Beijing, China

**Keywords:** Educational technology tools, LMS, Social networking systems, Student engagement, EFL courses

## Abstract

Educational technology has become an indispensable aspect of higher education, playing a crucial role in affecting student engagement, in particular. The application, advantages and disadvantages of learning management systems (LMS) and social networking systems in Chinese EFL courses, represented by Superstar—Xuexitong and WeChat, are introduced. The study aims to explore the relationship between the two educational technology tools and three dimensions of student engagement. Using an adopted and revised questionnaire from previous researches, the study measures the extent of impact of the specified educational technology tools on student engagement with the help of SPSS. Through the independent sample T tests, analysis of variance, correlation and regression, the study found that emotional engagement has the strongest positive effect on educational technology engagement. In addition, analysis of the four principal factors indicates that using LMS could engage students more than adopting social networking systems. There are significant differences in cognitive engagement between different genders, with that of males surpassing females. This article provides some empirical evidence for exploration into the use of educational technology in a specific discipline to foster better student engagement.

**Supplementary Information:**

The online version contains supplementary material available at 10.1186/s41239-021-00263-0.

## Introduction

The proliferation of digital technology has explicably attracted the researchers’ attention to the effectiveness and efficacy of technology in relation to learning and teaching processes and outcomes (Rashid & Asghar, 2016). Innovative information communication technology not only poses great challenges but also provide opportunities to teachers and students. Many such technology applications or tools are utilized as educational technology very effectively in schools. Challenges are manifested in several aspects: the update of teaching and learning hardware of higher educational institutions; the technological competence, including the mastery of computing skills and utility of different online resources; effective  integration of different modes of delivery, modes of teaching and styles of learning as a result of adopting a strategic and systematic approach to the use of technology combined with the best features of face-to-face interaction. However, the opportunities seem to be more appealing: better accessibility for teachers to teaching materials and students to learning materials; better facilitation for teachers to teaching management and formative assessment; the more diversified and efficient ways for teacher-student interaction; better student engagement and academic achievements.

Previous studies have already pointed out that student engagement is one determinant to a student’s academic success, cognitive development, and the quality of education (Hu & Kuh, 2002; Kahu & Nelson, 2017; Zhoc et al., 2019). The degree of engagement with academic-oriented activities is one of the key standards to measure student engagement level of learners. However, it’s also been discussed that the sole digital technology does not promote the students’ academic performance (Means et al., 2010). The main determinant lies in the proper integration of technology and pedagogy, therefore, the instructors play an important role in stimulating the students to be more engaged (Bond et al., 2019).

Therefore, calls have been made for a greater understanding of the role that educational technology plays in affecting student engagement, and the use of learning technology is an important concept that should fit into student engagement, in order to strengthen teaching practice and lead to improved outcomes for students (Bond et al., 2020; Krause & Coates, 2008; Nelson Laird & Kuh, 2005; Zhoc et al., 2019).

Various studies have found there is positive correlation between the use of technology and measures of engagement. Using information technology for educational purposes is linked to more general student engagement in effective educational practices, which is manifested in active and collaborative learning (Daniel Chen et al., 2010; Nelson Laird & Kuh, 2005; Sun et al., 2018). A survey done by Drain, Grier, and Sun finds there is a positive relationship between the time of educationally purposeful computer use and GPA, and educationally purposeful use of electronic devices is beneficial for academic performance (Drain et al., 2012). Results from a sample of Chinese junior secondary school students revealed the relationship between student engagement and motivation. Students were engaged in school when the instructional practices are motivating and they were social-emotionally supported (Lam et al., 2012).

### Student engagement and its three dimensions

Student engagement has been studied a lot by researchers all around the world (Bond & Bedenlier, 2019; Bond et al., 2020; Hu & Kuh, 2002). Engagement may refer to student and institutional level, academic and non-academic aspects of higher education experience. In this paper, the scope of activities in academic aspects is only taken into account, because educational technology is closely related to academic activities.

It is considered to be a complex construct involving multifaceted indicators such as participation, achievement, interaction etc., however, it is not sufficiently and properly defined, as a majority of the selected studies in the corpus of student engagement lack a definition, with only a small proportion of articles attempting to define the concept (Bond et al., 2020). According to Bond, engagement is defined as:

The energy and effort that students employ within their learning community, observable via any number of behavioral, cognitive or affective indicators across a continuum. It is shaped by a range of structural and internal influences, including the complex interplay of relationships, learning activities and the learning environment. The more students are engaged and empowered within their learning community, the more likely they are to channel that energy back into their learning, leading to a range of short- and long-term outcomes, that can likewise further fuel engagement.

It has been acknowledged that student engagement can be elaborated from three perspectives: behavioral engagement, cognitive engagement and emotional engagement (Fredricks et al., 2004, 2016). More recent studies broadened the concept of student engagement by adding one more angle, therefore, there are four perspectives of it: behavioral perspective, psychological perspective, sociocultural perspective and holistic perspective (Kahu, 2013). But this research is based on the typology of behavioral, cognitive and emotional engagement, as it is the most widely accepted and studied.

Behavioral engagement is made up of students’ involvement time, persistence to learning activities and efforts of participation (Bond et al., 2020; Miles & Stipek, 2006; Zhoc et al., 2019). There are different indicators to define behavioral engagement in previous studies: (1) by active conduct, such as time on tasks, class discussions; (2) by involvement in learning and academic tasks, such as attention and timely cooperation; (3) by previewing and reviewing activities (Wu & Zhang, 2018). However, most emphasized indicators are time on homework, class participation, participation in school-based activities and effort and academic interaction with peers, faculty and staff (Krause & Coates, 2008).

Cognitive engagement is related to intrinsic motivation, learning objectives, and self-regulation (Alioon & Delialioğlu, 2019; Ma et al., 2017), which corresponds with the study of Fredricks and his peers who believe the cognitive dimension of engagement most commonly refers to students’ self-regulation and effective use of deep learning strategies (Fredricks et al., 2004). It also involves learning strategies the students use in their learning activities. Indicators of cognitive engagement include deep learning (Fredricks et al., 2004), focus or concentration (Bond & Bedenlier, 2019) and positive self-perceptions and self-efficacy. Deep learning means the depth students study material, while self-regulation means how students prepare and seek information, and doing extra work and fulfilling more than the requirements of school (Henrie et al., 2015).

Emotional engagement is also known as affective engagement, which relates to positive reactions to the learning environment, peers and teachers, as well as their sense of belonging and interest (Bond & Bedenlier, 2019)^.^ Motivation can help students to achieve high grades or a qualification, or feel pleasure and interest in their learning (Kahu, 2013). The result from 243 studies by Bond and other researchers revealed top five most often identified affective student engagement indicators according to student engagement dimension: positive interaction with teachers and peers, enjoyment, positive attitude about learning/Interest, motivation and enthusiasm (Bond et al., 2020).

### Educational technology in EFL courses

What educators are facing today are increasingly emerging educational technology tools and the proper incorporation of the suitable technology tools into their present teaching. In order to achieve better teaching and learning outcomes, the educators need to know all the merits and drawbacks of the technology tools they and their students could have access to.

Educational technology tools are prevalently used in Chinese EFL courses. To be specific, there are learning managing systems (LMS), social networking systems, assessment tools and all sorts of software, among which LMS and social networking systems have been prominently used by educators and practitioners. In addition, the two types of educational technology presumably have different effects on student engagement, therefore, the purpose is to investigate what effect of each of the two technology tools on three dimensions of student engagement, respectively.

### Learning management systems (LMS)

The maturity of LMS occurred in the 2000s and worldwide adoption of the educational technology at universities has had great impact on teachers, students and administers. A large-scale 2014 survey in the United States indicated that 99% of educational institutions support at least one learning management system. However, even though 74% of the faculty use it and 71% consider it useful for the teaching and learning, the faculty seldom use advanced features of LMS (Anderson & Dron, 2016). The analysis and results by EDUCAUSE on 113,035 students in 251 universities in 13 countries, revealed that official websites of the institutions and LMS are the most valuable platform, which play a significant role in the utmost success of students (Li & Zhou, 2018). More researches reveal the universality and usefulness of LMS (Henderson et al., 2017). Meanwhile, the demonstration of knowledge of LMS was identified as poorly prepared and more advanced features were not fully made advantage of (Bond et al., 2018).

The most prevalent LMS among Chinese universities include Superstar—Xuexitong, RainClassroom—Yuketang, Tsinghua Educational Online (THEOL), to name a few. Many LMS have been introduced, tried and adopted by teachers in their teaching or research, however, the scale has never been spread to almost every corner of education until the breakout of COVID-19. Since Spring of 2020, with the implementation of online teaching across the nation, various LMS have been widely used in all levels of schools. Xuexitong has been undergoing tremendous challenges, improvement and updating in order to meet the unprecedented need from institutions, faculty and students. Originally, the LMS was merely used to upload  syllabuses, course content, student name lists and mainly as a subsidiary educational tool to the face-to-face teaching. All of a sudden, teachers find that various class activities can be organized and achieved through different modes of functions. They can use the LMS to take attendance, assess the students’ performance, grade assignments, hold quizzes and give feedback, etc. Besides, PBL and topic discussions can be conveniently designed and generally welcomed.

The relationship between Twitter, LMS and student engagement has been studied, with the result that Twitter usage positively affected student engagement at the course level and LMS usage had a positive relationship with student engagement at the school level (Williams & Whiting, 2016). Nevertheless, the focus is not on the three dimensions of student engagement, thereby, no further research has been carried out on the indicators as so to draw more explicit conclusions on promoting the index.

### Social networking systems

Social networking systems have been actively introduced to EFL courses since their invention. The prominent systems include blog, microblog, Fetion (outdated now), Tencent QQ and WeChat. Educators establish groups with their students and make good use of the functions, such as notifying, sharing content, voting or communicating academically or non-academically. Social networking systems are the most convenient because they are the most accessible tool, enabling posting comments, pictures, audios or videos. However, there is the limitation of content volume. In EFL courses, WeChat groups are also often used by faculty and students to communicate, ask and answer questions concerning English studies. WeChat is indispensable to the virtual teacher–student communication.

Various studies examine the effect of Online Social Networks (OSNs), namely social  networking systems in this paper, on student academic performance. Correspondingly, a myriad of advantages of Facebook used as an educational tool has been summarized, such as being a valuable platform to promote student engagement and developing an emotional connection and a stronger sense of community (Chugh & Ruhi, 2018). On the condition that the students use Facebook as an educational tool, desirable results in knowledge sharing and discussion was achieved (Masrom et al. 2021). In order to further explore the effect of teacher facilitation on students’ learning performance by using WeChat, Xu et al. (2020) conducted a quasi-experiment between experiment groups of students (with teacher facilitation) and control groups (without teacher facilitation), and came to the conclusion that behavioral and cognitive engagement were different while emotional engagement was not affected much.

During the process of exploring and employing different educational technology tools, student engagement has been researched tremendously. And the three aspects have also been discussed sufficiently, with consensus being reached that the instruction of educators plays a significant role in promoting student engagement (Ma et al., 2015). Currently, the reasonable design of instruction and online learning, the scientific incorporation of all the present resources that an instructor is disposed of and the ratio of face-to-face instruction and after-class online learning has aroused great attention and discussions among researchers. With the popularity of so many convenient and advanced educational technology, this study focuses on the following research questions:

RQ1: What is the relationship between three dimensions of student engagement and educational technology engagement?

RQ2: What’s the relationship between LMS with three dimensions of student engagement in EFL courses? What about social networking systems and the three dimensions of student engagement in the same context?

RQ3: Does the effect on student engagement differentiate in grades, genders and majors of the students?

## Methods

### Participants

Participants are 268 undergraduates and graduates enrolling in EFL courses (College English, Advanced Oral English, Academic English) at a university in Beijing, China. EFL courses are compulsory for the undergraduates and graduates who are supposed to learn it for four semesters, 16 weeks each semester. But seniors don’t have to take EFL courses since they are supposed to put more efforts in dissertation writing and hunting a job. The undergraduates include freshmen who have already taken EFL courses for one semester, sophomores (three semesters), juniors (five semesters) and graduates. Among them, there are 135 males (50.4%) and 133 females (49.6%), and sophomores account for the largest proportion (37.7%) while the juniors the second largest (21.6%). The mean age of the respondents is 20.8 years old. From the first year of their English learning, they were exposed to various educational technology tools. All the participants are quite familiar with the application of educational technology tools. 66% of the respondents (n = 177) major in Science and Engineering while 34% (n = 91) major in Liberal & Arts. When asked to choose the educational technology they use more in their EFL courses, the majority of participants (n = 173; 64.6%) say they prefer LMS.

### Measurement scale

The following questionnaire (see Additional file 1: Appendix S1) was designed based on the three dimensions of student engagement: behavioral, cognitive and emotional, with educational technology SE as the fourth category. It was adapted from “Factor Loadings for Core Survey Items and Technology Items” (22 items) and “Engagement with Information Technology Scales” (18 items) by Nelson Laird and Kuh (2005), Higher Education Student Engagement Scale (HESES; 28 items), and Items Comprising the Seven-Factor Model and Standardized Loadings (Moreira et al., 2020; 60 items). The items were carefully chosen by the authors, and revised three times by a psychologist expert according to need of the current research.

After a respondent finishes answering the basic information, he or she needs to choose an option between social networking systems and leaning management systems, then the questionnaire will orient to the corresponding scale. Since the items are originally in English while the respondents are Chinese college students, all the statements are bilingual (English and Chinese) for better clarity and understanding. This survey was trialed by the expert, the researchers and a group of students who attended the same course by the second researcher, in order to test its credibility and for further revision and perfection. The T-test shows that the survey is scientific and valid. The scale is adopted Likert 1 = strongly disagree, 2 = disagree, 3 = neutral, 4 = agree, 5 = strongly agree.

### Data collected

The target respondents include not only undergraduates but graduates who have EEL courses presently. The survey was carried out with the respondents anonymously. The questionnaire was designed on a free platform specifically for survey, tests and voting, and then distributed to the students electronically by WeChat. In order to collect sufficient samples and motivate the participants to take the survey seriously, a minor bonus was set up with the questionnaire. After agreeing to participate, students read the instructions and completed the questionnaire. The data was analyzed in SPSS to answer the research questions. First, the descriptive statistics were conducted to initially explore the data. In order to test the inter-consistency of the questionnaire, we did a reliability analysis. The result revealed that Cronbach’s Alpha of behavioral engagement, cognitive engagement and emotion engagement is 0.939, 0.960, 0.939 respectively, and that of Educational technology engagement is 0.950, with that of the whole scale being 0.974, which proves to its good reliability (Table 1).

**Table 1:** Reliability statistics

	Cronbach’s alpha	N of items
Behavioral engagement	0.939	8
Cognitive engagement	0.960	8
Emotional engagement	0.939	8
Educational technology engagement	0.950	10
Total	0.974	34

## Results

### Descriptive statistics

The mean and standard deviation of each of the four factors was obtained. Results show that Kaiser–Meyer–Olkin measure of sampling adequacy is 0.957 and the significance of Bartlett’s test of sphericity is 0.000 < 0.001, which indicates the reliability of the principal component analysis and the compactness of the correlations to produce distinct components (see Table 2).

**Table 2:** KMO and Bartlett’s test

Kaiser–Meyer–Olkin measure of sampling adequacy	.957
Bartlett’s test of sphericity	
Approx. Chi-square	9617.490
df	561
Sig	.000

Principal component factor analysis shows that four components with initial features greater than 1 were extracted. The total variance cumulative of first component (cognitive engagement) is 21.355%, the total variance cumulative of the second component (educational technology engagement) is 19.725%, that of the third component (behavioral engagement) is 17.861% and the fourth (emotional engagement) is 14.352%. The final total variance cumulative reaches 73.294%, which demonstrates that the extracted components could explain most of the questions (see Table 3).

**Table 3:** Total variance explained

Component	Initial eigenvalues			Extraction sums of squared loadings			Rotation sums of squared loadings		
	Total	% of variance	Cumulative %	Total	% of Variance	Cumulative %	Total	% of variance	Cumulative %
1	18.653	54.862	54.862	18.653	54.862	54.862	7.261	21.355	21.355
2	2.716	7.988	62.850	2.716	7.988	62.850	6.707	19.725	41.081
3	2.199	6.469	69.319	2.199	6.469	69.319	6.073	17.861	58.942
4	1.352	3.976	73.294	1.352	3.976	73.294	4.880	14.352	73.294

Extraction method: principal component analysis

1 = Cognitive engagement, 2 = Educational technology engagement, 3 = Behavioral engagement, 4 = Emotional engagement

All the items are categorized into four factors: cognitive engagement, educational technology engagement, behavioral engagement and emotional engagement. There are 8 items for the three engagement dimensions (cognitive, behavioral, emotional) respectively, and 10 items for educational technology engagement, so in total there are 34 items. All the items correspond the top five most identified student engagement indicators according to engagement dimension in a corpus of 243 studies. Specifically, according to the pedagogical characteristics of EFL courses such as tremendous interaction, participation and practice, the behavioral SE items involve participation/interaction/involvement, study habits, responsibility; the cognitive SE items include deep learning, self-regulation, positive self-perception; the emotional SE items correspond with positive interaction with teachers and peers, enjoyment, positive attitude about learning, interest, and sense of belonging (Bond & Bedenlier, 2019). It’s not an easy process to categorize so many items related to student engagement. When it comes to the educational technology  engagement items, the researchers design two scales, with the first 7 items identical while the last three vary. For social networking  systems, online discussion, contact with friends and contact with lecturers/tutors are adopted, since the communication function is more obvious for it. For LMS, online subjects, learning online resources at one’s own pace and specifically designed web-based resources and information are adopted, since the platform is more suitable for these tasks.

### Statistical analyses

RQ1: What is the relationship between three dimensions of student engagement and educational technology engagement?

In order to determine the correlation between the three dimensions of student engagement and educational technology engagement, we did correlation analysis. The results show that there is significant correlation between behavioral engagement, cognitive engagement, emotional engagement and educational technology engagement (p < 0.05), which means that strong student engagement has positive effect on educational technology engagement (see Table 4). To be specific, the items of cognitive engagement such as “My education will create many opportunities for me”, “I am hopeful about my future” and “Learning is fun because I get better at something” (items 6, 7, 8), corresponding with investment in learning and positive self-perception, has the biggest influence among the eight. Items of behavioral engagement such as “Rarely skip classes”, “Usually come to class having completed readings or assignments” and “Ask questions in class or contributed to class discussions” (items 5, 6, 1) score the highest, manifesting that attendance, participation and involvement are the most obvious indicators. Items of emotional engagement such as “Have serious conversations with students who are very different from me”, “Feel part of a group of students committed to learning” and “Really like being on my campus” (items 4, 6, 8) rank on the top, standing for positive interactions with peers, sense of connectedness and belonging. These indicators most significantly lead to better educational technology engagement.

**Table 4:** Correlations between behavioral engagement, cognitive engagement, emotional engagement and educational technology engagement

	Behavioral engagement	Cognitive engagement	Emotional engagement	Educational technology engagement
Behavioral engagement				
Pearson correlation	1			
Sig. (2-tailed)				
Cognitive engagement				
Pearson correlation	.578^**^	1		
Sig. (2-tailed)	.000			
Emotional engagement				
Pearson correlation	.630^**^	.735^**^	1	
Sig. (2-tailed)	.000	.000		
Educational technology engagement				
Pearson correlation	.615^**^	.705^**^	.799^**^	1
Sig. (2-tailed)	.000	.000	.000	

^**^Correlation is significant at the 0.01 level (2-tailed)

In order to further explore the effect of the three student engagement dimensions on educational technology engagement, we did regression analysis. The result showed that the Adjusted R Square is 68.0% and the model fit is quite good, so the overall model is significant (F = 187.137, p < 0.05). The three dimensions of student engagement are the predictors (constant), while educational technology engagement is the dependent variable (see Table 5).

**Table 5:** ANOVA

Model	Sum of squares	df	Mean square	F	Sig
1					
Regression	105.105	3	35.035	187.137	.000^a^
Residual	49.425	264	.187		
Total	154.529	267			

Dependent variable: educational technology engagement

^a^Predictors: (constant), emotional engagement, behavioral engagement, cognitive engagement

Table 6 illustrates the summary effects of the three primary student engagement components on educational technology engagement. Coefficient of behavioral engagement on educational technology engagement is 0.122 (p = 0.002 < 0.05), so behavioral engagement is significantly positive to educational technology engagement. The same effects can also be found with Cognitive engagement and Emotional engagement (both at p = 0.000 < 0.05). Among the three basic engagement types, emotional engagement is the most significant factor with the coefficient of 0.518, which means the emotional involvement can strongly affect the Educational technology engagement of the users.

**Table 6:** Regression analysis results of three engagement dimensions on educational technology engagement

Model	Unstandardized coefficients		Standardized coefficients	t	Sig
	B	Std. error	Beta		
1					
(Constant)	.815	.131		6.223	.000
Behavioral engagement	.122	.039	.144	3.143	.002
Cognitive engagement	.193	.046	.221	4.202	.000
Emotional engagement	.518	.052	.545	9.868	.000

^a^Dependent variable: educational technology engagement

RQ2: What’s the relationship between LMS with three dimensions of student engagement in EFL courses? What about social networking systems and the three dimensions of student engagement in the same context?

The researchers would like to probe whether there are variances between the three dimensions of engagement and educational technology engagement in social networking systems and LMS, so independent samples test was done to answer research question 2. The number of respondents who choose social networking systems is 95, while the number of LMS is 173, which is almost twice the number of the former. It is completely out of the expectation of the researchers. Usually, students use more WeChat in their daily life so their involvement and time spend on it is striking, and teachers would consider it the primary educational technology when it comes to EFL courses. The fact proves that LMS surpasses social networking systems in enhancing student engagement.

The results (Table 7) reveal that there are significant differences between the two educational technology tools (p < 0.05). To be precise, the mean values of four engagement aspects of LMS are obviously higher than those of the social networking systems. The mean values of behavioral engagement of LMS and social networking systems are 3.8620 and 3.3437, cognitive engagement are 3.5000 and 3.2763, emotional engagement are 3.7936 and 3.3687, educational technology engagement are 3.9416 and 3.5768 respectively. Especially, LMS represents more engagement in behavioral and emotional dimensions. Presumably, Xuexitong facilitates the students to “receive prompt feedback from faculty on my academic performance” and requires students to “spend a lot of time studying on my own” (Behavioral engagement items 2 and 4). Besides, the students use LMS as the inclusive platform to learn EFL courses, they have strong feelings of community and sense of belonging (Cognitive engagement items 6, 7, 8). By contrast, WeChat has almost become the top application on one’s cell phone and it has powerful reminder function, which attracts the students to pay extra attention to it, even though during the process of learning, thus decreasing their concentration, efficiency and engagement.

**Table 7:** Independent samples test of three dimensions of engagement and educational technology engagement

	Levene’s test for equality of variances		t-test for equality of means						
	F	Sig	t	df	Sig. (2-tailed)	Mean difference	Std. error difference	95% confidence interval of the difference	
								Lower	Upper
Behavioral engagement									
Equal variances assumed	1.622	.204	− 4.696	266	.000	− .51834	.11038	− .73567	− .30100
Cognitive engagement									
Equal variances assumed	1.232	.268	− 2.028	266	.044	− .22368	.11032	− .44090	− .00646
Emotional engagement									
Equal variances assumed	.000	.986	− 4.283	266	.000	− .42485	.09919	− .62014	− .22955
Educational technology engagement									
Equal variances assumed	1.078	.300	− 3.851	266	.000	− .36478	.09473	− .55128	− .17827

RQ3: Does the four types of student engagement differentiate in genders, grades and majors of the students?

Results of independent sample test of different genders (Table 8) indicate that there are no significant differences between genders when measuring behavioral, emotional and educational technology engagement (p > 0.05), however, the mean value (= 3.5293) of cognitive engagement of males is slightly significantly higher than that of females (mean = 3.3105). The level of cognitive engagement stands for the investment in learning and it is a psychological commitment to mastering the knowledge and skills (Lan & Hew, 2020). From the finding, we may assume that the male students have a better understanding of the subject content. From the items, we can conclude that male students also have better strategies to manage their academic workload, higher integration ability and put in more endeavors.

**Table 8:** Independent samples test of different genders

	Levene’s test for equality of variances		t-test for equality of means						
	F	Sig	t	df	Sig. (2-tailed)	Mean difference	Std. error difference	95% confidence interval of the difference	
								Lower	Upper
Behavioral engagement									
Equal variances assumed	3.409	.066	− .475	266	.635	− .05222	.10985	− .26850	.16407
Cognitive engagement									
Equal variances assumed	7.891	.005	2.073	266	.039	.21873	.10551	.01098	.42648
Emotional engagement									
Equal variances assumed	1.789	.182	.918	266	.359	.08996	.09796	− .10291	.28283
Educational technology engagement									
Equal variances assumed	4.073	.045	.808	266	.420	.07519	.09301	− .10793	.25831

Meanwhile, more independent sample tests were done concerning different grades and majors, however, the results were quite disappointing since there were no significant differences found for the respondents from different grades or majors (p > 0.05). Thus, no matter what grades or majors the students are in, the engagement they show in EFL courses do not vary prominently. What matters is the variance between using different educational technology tools, which can be a general rule applicable in teaching.

## Conclusions and discussion

The current study takes educational technology engagement as a new dimension of the student engagement construct. By adopting and revising previous scales, the paper produces a new student engagement scale with educational technology engagement as a highlight, because of the prevalent use and pervasive influence of it, this facet cannot be neglected. The two educational technology tools are so frequently used that it is imperative to find out their differences and similarities on impacting student engagement so as to enhance academic performances of students.

Thus, the study explores how the two educational technology tools influence student engagement in EFL courses in a Chinese university. According to the data, the research found that the three dimensions of student engagement all have significant effect on educational technology engagement, while emotional engagement has the biggest impact among the three. This corresponds with the revelation that general student engagement factor is positively correlated with emotional well-being (Moreira et al., 2020). Findings from our research provide preliminary support for utilizing educational technology as a positive factor influencing student engagement.

Conversely, the two types of educational technology both have significant influence on tridimensionality of student engagement, which may lead to more investment in terms of academic outcomes. More specifically, LMS influences student engagement more significantly, surpassing the social networking systems in this research. Just as found in the systematic review, social networking tools are more disengaging than engaging (Bond et al., 2020). Therefore, instructors should continue to use LMS more frequently and strategically in their teaching, so that it could exert better influences on student engagement. Besides assigning video-watching, quiz-taking and even video conferencing, LMS also serves as an effective platform for teachers to hold discussion activities and students to share their ideas or views. Besides, the group task part is also quite popular among all the functions, since students can upload their after-class project videos and be peer-reviewed by other members. Accordingly, it’s advised that self-directed learning, online assignments and online assessment should be properly arranged while employing LMS, in order to make best use of the resources.

However, what cannot be denied is the problems emerging from LMS. Take Xuexitong for an example, it is so comprehensive that it seems to be adopted by teachers in all disciplines. As a result, it cannot be so specifically targeted to satisfy all kinds of needs of teachers and students. For example, the system does not apply to assessment of students’ oral English ability since it is not so professionally designed, and the teachers have to switch to another course-related educational technology tool to fulfill this. On the other hand, Xuexitong also requires the teachers’ good computer efficacy to get familiar with multifunction features, which definitely takes time and efforts and thus adds burden to teachers.

On the other hand, efforts should also be paid to the design and pedagogical use of social networking systems, from the teachers’ perspective. Schindler and his peers reviewed studies of twitter—one prominent social networking systems and student engagement, with mixed findings. Course-specific use of the tool without having to create an account resulted in much higher participation rates, furthermore, the required and integrated use into class discussions brought 100% participation rates (Schindler et al., 2017). Researchers also found evidence of student–student and student-instructor interactions by using Twitter (Hennessy et al., 2016). These previous studies also imply similar application of WeChat in EFL courses by asking students to post updates, ask and answer questions and sharing resources. Besides, the use of WeChat should be monitored and guided when students are having class, otherwise they would easily get distracted either by new coming of the short messages or unconsciously indulge themselves in browsing circles or short videos. It illustrates the relatively lower involvement by using this technology in EFL courses.

## Limitations

Other researchers found that use of technology has a stronger impact earlier in the college experience. The present study has already increased the sample size and include students from different years, more majors, educational institutions and backgrounds in order to enhance the representativeness of the sample. Although participants in the study represented educational technology-mediated students, our samples are still homogeneous culturally and socially, so our further research aiming to include the overseas students in the university, or more universities locally or nationally.

Previous studies have made some researches on the relationship between information technology and student engagement (Nelson Laird & Kuh, 2005), however, this research focuses on two types of educational technology which are very closely related to the students’ EFL learning, so the study is more specific and thus the instructors and students would be more aware of the usages of educational technology. In addition, few have taken the genders into consideration when the three-dimension engagement is involved. Therefore, this research has provided empirical evidence to show that cognitive engagement differentiates significantly in males and females, with the mean value of males higher than that of females. In the meanwhile, behavioral, emotional and educational technology engagement does not impact males or females differentially.

The positive correlation between the use of educational technology and measure of engagement replicates the findings of the studies by Hu and Kuh (2002), Nelson Laird and Kuh (2005). They assert that engagement with information technology is associated with active and collaborative learning, and student-faculty interaction (Nelson Laird & Kuh, 2005), apparent indicators of behavioral engagement. The same relationship is reaffirmed by Pu-Shih Daniel Chen et al. whose results show a general positive relationship between the use of learning technology and student engagement and learning outcomes.

The measure scales are scrutinized from several existing student engagement scale pools, according to indicators of the three dimensions, however, some items are still ambiguous and hard to be undoubtedly categorized. Furthermore, the educational technology engagement may not be totally validated because it is adopted and revised from two or three studies concerning information technology, which is broader than educational technology. From the survey results, the researchers realize three of the items (indicating the differences between social networking systems and LMS) in educational technology engagement should have been rearranged so that their distinctions can be more evident.

Students showing more engagement in LMS implies that instructors need to make more efforts to draw out bigger potential of this platform by redesigning their courses and smart using, so that the students could be more involved. Suggestions are put forward from the three dimensions of student engagement: more interest should be aroused; more interactive activities should be added to EFL courses to increase the students’ active participation; more specific learning planning should be provided so that students may have better learning goals to be more engaged.

## Supplementary Information

**Additional file 1: Appendix S1.** Questionnaire. **Appendix S2.** Rotated component matrix of factor loading.

## Data Availability

Not applicable.
