# Supplementary material for: The effect of two educational technology tools on student engagement in Chinese EFL courses
Source: Int J Educ Technol High Educ. 2021 May 28;18(1):27. doi: 10.1186/s41239-021-00263-0 (PMC8159523; doi:10.1186/s41239-021-00263-0)
Supplement: Supplementary file 1 — Additional file 1: Appendix S1. Questionnaire. Appendix S2. Rotated component matrix of factor loading. [file 41239_2021_263_MOESM1_ESM.docx]

**Appendix 1:**

**Questionnaire**

**Which educational technology do you use more in your EFL learning? 在大学英语学习中，更多地使用以下哪种教育技术？**

**Social networking systems社交网络系统（微信、QQ等）(Direct to Educational technology engagement—Social network system跳转到教育技术投入--社交网络系统)**

**Learning management systems学习管理系统（学习通、雨课堂等）(Direct to Educational technology engagement—Learning Management Systems跳转到教育技术投入—学习管理系统)**

**Behavioral Engagement行为投入**

1. Ask questions in class or contributed to class discussions在课堂上提问或者参与课堂讨论
2. Receive prompt feedback from faculty on my academic performance 收到教师对我学习成绩的及时反馈
3. Regularly study on the weekends经常在周末学习
4. Spend a lot of time studying on my own自己花费大量时间学习
5. Rarely skip classes很少逃课
6. Usually come to class having completed readings or assignments通常在完成阅读或作业之后来上课
7. Regularly work with other students on course areas I have problems经常与其他学生一起学习我遇到问题的课程
8. Regularly get together with other students to discuss courses定期与其他学生聚在一起讨论课程

**Cognitive engagement认知投入**

1. Strategic about the way I manage my academic workload 运用策略去管理学业负担
2. Put together ideas or concepts from different courses when completing assignments 完成作业时能够整合不同学科的想法或概念
3. Worked harder than I thought I could to meet an instructor’s standards 我的努力程度超过教师的要求
4. Enjoy the intellectual challenge of courses studying喜欢课程学习带来的智力挑战
5. Finding my courses intellectually stimulating发现我的课程在智力上有激励性
6. My education will create many future opportunities for me教育会为我的未来创造很多机会
7. I am hopeful about my future我对未来充满信心
8. Learning is fun because I get better at something 学习很有趣因为我在某方面有所提高

**Emotional engagement情感投入**

1. I am interested in the work I get to do in my classes 我对课堂上要做的事情感兴趣
2. I feel excited by the work in my school 我对学校事务感到兴奋
3. Talk about career plans with a faculty member or advisor 与教师或辅导员讨论职业规划
4. Have serious conversations with students who are very different from me和与自己迥然不同的同学认真交谈
5. Include diverse perspectives in class discussions or writing assignments在课堂讨论或写作任务中纳入不同观点
6. Feel part of a group of students committed to learning 感觉自己属于一个热爱学习的团体
7. Really like being a university student 喜欢当一个大学生
8. Really like being on my campus喜欢我的校园

**Educational technology engagement—Social network system社交网络系统**

1. My instructors frequently require me to use educational technology to complete course assignments老师经常要求我使用此教育技术完成课程任务
2. Frequently communicate with classmates online to complete academic work经常与同学网上交流完成作业
3. Frequently work in teams outside of class using educational technology to complete course assignments.课外经常和小组同学一起使用此教育技术来完成课程任务
4. My instructors frequently use educational technology我的老师经常使用此教育技术
5. Gain new insights into course material from online discussions 网上讨论让我对课程内容有了深入了解
6. Online resources (e.g. course notes, free software and materials on the web) are very useful for me 网上资源（比如课程笔记、网上的免费软件和其他材料）对我非常有用
7. Regularly use the educational technology to contact friends in my course定期使用此教育技术与同修本门课程的朋友联系
8. Regularly use the educational technology for study purpose定期为了学习目的使用此教育技术
9. Regularly use online discussion groups related to my study经常使用与学习相关的网上讨论小组
10. Regular use this educational technology to contact lecturers/ tutors经常使用此教育技术联系授课老师或导师

**Educational technology engagement—Learning Management Systems (LMS)学习管理系统**

1. My instructors frequently require me to use educational technology to complete course assignments老师经常要求我使用此教育技术完成课程任务
2. Frequently work in teams outside of class using educational technology to complete course assignments.课外经常和小组同学一起使用此教育技术来完成课程任务
3. My instructors frequently use educational technology我的老师经常使用此教育技术
4. Gain new insights into course material from online discussions 网上讨论让我对课程内容有了深入了解
5. Frequently take subjects offered online with no face-to-face classes经常学习非面授的网上课程
6. Online resources (e.g. course notes, free software and materials on the web) are very useful for me 网上资源（比如课程笔记、网上的免费软件和其他材料）对我非常有用
7. Frequently learn at my own pace using online resources经常按照自己的节奏使用网上资源学习
8. Regularly use web-based resources and information designed specifically for this course定期使用专门为这门课程设计的网络资源和信息
9. Regularly use online discussion groups related to my study经常使用与学习相关的网上讨论小组
10. Regularly use the educational technology for study purpose定期为了学习目的使用此教育技术

**Appendix 2**

| Rotated Component Matrix^a^ of Factor Loading | | | | |
| --- | --- | --- | --- | --- |
|  | Component | | | |
|  | 1 | 2 | 3 | 4 |
| Cognitive engagement 6 | .815 |  |  |  |
| Cognitive engagement 7 | .811 |  |  |  |
| Cognitive engagement 8 | .800 |  |  |  |
| Cognitive engagement 5 | .794 |  |  |  |
| Cognitive engagement 2 | .787 |  |  |  |
| Cognitive engagement 1 | .775 |  |  |  |
| Cognitive engagement 4 | .762 |  |  |  |
| Cognitive engagement 3 | .701 |  |  |  |
| Educational technology engagement 8 |  | .777 |  |  |
| Educational technology engagement 10 |  | .775 |  |  |
| Educational technology engagement 9 |  | .748 |  |  |
| Educational technology engagement 3 |  | .733 |  |  |
| Educational technology engagement 6 |  | .694 |  |  |
| Educational technology engagement 5 |  | .675 |  |  |
| Educational technology engagement 1 |  | .667 |  |  |
| Educational technology engagement 2 |  | .621 |  |  |
| Educational technology engagement 4 |  | .604 |  |  |
| Educational technology engagement 7 |  | .553 |  |  |
| Behavioral Engagement 5 |  |  | .810 |  |
| Behavioral Engagement 6 |  |  | .810 |  |
| Behavioral Engagement 1 |  |  | .793 |  |
| Behavioral Engagement 7 |  |  | .772 |  |
| Behavioral Engagement 8 |  |  | .772 |  |
| Behavioral Engagement 3 |  |  | .735 |  |
| Behavioral Engagement 2 |  |  | .728 |  |
| Behavioral Engagement 4 |  |  | .658 |  |
| Emotional engagement 4 |  |  |  | .753 |
| Emotional engagement 6 |  |  |  | .747 |
| Emotional engagement 8 |  |  |  | .738 |
| Emotional engagement 7 |  |  |  | .715 |
| Emotional engagement 3 |  |  |  | .609 |
| Emotional engagement 5 |  |  |  | .523 |
| Emotional engagement 2 |  |  |  | .520 |
| Emotional engagement 1 |  |  |  | .520 |
| Extraction Method: Principal Component Analysis.  Rotation Method: Varimax with Kaiser Normalization. | | | | |
| a. Rotation converged in 6 iterations. | | | | |
